# miRNA signature associated with outcome of gastric cancer patients following chemotherapy

**DOI:** 10.1186/1755-8794-4-79

**Published:** 2011-11-23

**Authors:** Chang Hee Kim, Hark K Kim, R Luke Rettig, Joseph Kim, Eunbyul T Lee, Olga Aprelikova, Il J Choi, David J Munroe, Jeffrey E Green

**Affiliations:** 1 Advanced Technology Program, SAIC-Frederick, Inc., National Cancer Institute-Frederick, Frederick, MD 21701, USA; 2 Laboratory of Cancer Biology and Genetics, National Cancer Institute, Bethesda, MD 20892, USA; 3 National Cancer Center, Goyang, Gyeonggi, Republic of Korea, 410-769

## Abstract

**Background:**

Identification of patients who likely will or will not benefit from cytotoxic chemotherapy through the use of biomarkers could greatly improve clinical management by better defining appropriate treatment options for patients. microRNAs may be potentially useful biomarkers that help guide individualized therapy for cancer because microRNA expression is dysregulated in cancer. In order to identify miRNA signatures for gastric cancer and for predicting clinical resistance to cisplatin/fluorouracil (CF) chemotherapy, a comprehensive miRNA microarray analysis was performed using endoscopic biopsy samples.

**Methods:**

Biopsy samples were collected prior to chemotherapy from 90 gastric cancer patients treated with CF and from 34 healthy volunteers. At the time of disease progression, post-treatment samples were additionally collected from 8 clinical responders. miRNA expression was determined using a custom-designed Agilent microarray. In order to identify a miRNA signature for chemotherapy resistance, we correlated miRNA expression levels with the time to progression (TTP) of disease after CF therapy.

**Results:**

A miRNA signature distinguishing gastric cancer from normal stomach epithelium was identified. 30 miRNAs were significantly inversely correlated with TTP whereas 28 miRNAs were significantly positively correlated with TTP of 82 cancer patients (*P*<0.05). Prominent among the upregulated miRNAs associated with chemosensitivity were miRNAs known to regulate apoptosis, including let-7g, miR-342, miR-16, miR-181, miR-1, and miR-34. When this 58-miRNA predictor was applied to a separate set of pre- and post-treatment tumor samples from the 8 clinical responders, all of the 8 pre-treatment samples were correctly predicted as low-risk, whereas samples from the post-treatment tumors that developed chemoresistance were predicted to be in the high-risk category by the 58 miRNA signature, suggesting that selection for the expression of these miRNAs occurred as chemoresistance arose.

**Conclusions:**

We have identified 1) a miRNA expression signature that distinguishes gastric cancer from normal stomach epithelium from healthy volunteers, and 2) a chemoreresistance miRNA expression signature that is correlated with TTP after CF therapy. The chemoresistance miRNA expression signature includes several miRNAs previously shown to regulate apoptosis *in vitro*, and warrants further validation.

## Background

miRNAs are short (~22 nucleotide), non-coding RNAs that regulate gene expression primarily by translational repression or transcriptional degradation [1]. miRNAs have great potential as cancer biomarkers because of their tissue-specific expression and their aberrant expression in cancer cells [2]. Additionally, miRNAs have important functions in cell cycle regulation and apoptosis. The expression of miRNAs may be dysregulated in cancer by a variety of mechanisms including transcriptional regulation, amplification, deletion, mutation, and epigenetic silencing [3]. Thus, microRNAs may be potentially useful biomarkers that help guide individualized therapy.

Identifying patients who likely will or will not benefit from cytotoxic chemotherapy through the use of biomarkers could greatly improve clinical management by better defining appropriate treatment options for patients. Most previous studies attempting to identify miRNA predictors of chemoresistance in cancer have examined only individual miRNAs [4]. Thus far, only one published high-throughput microarray analysis has evaluated miRNA expression signatures as predictors of chemotherapy resistance in metastatic solid tumor patients [5]. In this miRNA microarray study of stage III-IV ovarian cancers, let-7i expression was found to be significantly reduced in 27 chemotherapy-resistant patients as compared to 42 complete responders, although there was no independent validation cohort [5].

Here we present the results of a prospective study utilizing a high-throughput miRNA microarray analysis in which a miRNA expression signature has been identified that distinguishes gastric cancer from normal stomach epithelium. Further, we have identified a second signature that is correlated with the time to progression (TTP) for gastric cancer patients treated with cisplatin and fluorouracil (CF), a reference chemotherapy regime for gastric cancer. These miRNA signatures may be useful as potential biomarkers to help in the diagnosis of gastric cancer in difficult cases and to predict response of gastric cancer patients to CF therapy.

## Results

### Identification of a gastric cancer miRNA signature

Ninety pretreatment gastric cancer tissue samples were available for this analysis and their clinicopathological characteristics are described in Table 1. All patients had metastatic disease at the time of enrollment and after endoscopic biopsy tissue samples were collected, the patients were treated with cisplatin and fluorouracil (or capecitabine) combination chemotherapy. All microarray data has been deposited at GEO and is available upon publication. Reviewer access: http://www.ncbi.nlm.nih.gov/geo/query/acc.cgi?token=ftixhsoiemwgyfi&acc=GSE30070

**Table 1:** Clinico-pathological characteristics of patients

	*Gastric cancer patient*		*Healthy volunteer*
	Training set	Proof-of-principle test set (responder)	
***Number***	82	8	34
***Age - yr***			
Median	56	56	48
Interquartile range	(44-63)	(44-58)	(43-57)
***Sex - no. (%) ***			
Male	64 (78.0%)	7 (87.5%)	23 (67.6%)
Female	18 (22.0%)	1 (12.5%)	11 (32.4%)
***Performance status (PS) - no. (%)***			
ECOG^1 ^PS 0 or 1	73 (89.0%)	8 (100%)	
ECOG PS 2 or 3	9 (11.0%)	0	
***Histological type - no. (%)***			
Lauren's intestinal	34 (41.5%)	3 (37.5%)	
Lauren's diffuse	48 (58.5%)	5 (62.5%)	
***Location of primary lesion - no. (%)***			
Upper 1/3	11 (13.4%)	1 (12.5%)	
Middle 1/3	18 (22.0%)	5 (62.5%)	
Lower 1/3	43 (52.4%)	1 (12.5%)	
Entire stomach	10 (12.2%)	1 (12.5%)	
***Chemotherapy regimen - no. (%)***			
Cisplatin/Fluorouracil	80 (97.6%)	8 (100%)	
Cisplatin/Capecitabine	2 (2.4%)	0	
*Relative dose intensity - %			
Median	81.2	76.6	
Interquartile range	(75.3-87.3)	(64.7-84.9)	
***Number of chemotherapy cycles ***			
Median	4	10	
Interquartile range	(2-5)	(7-11)	
***Chemotherapy response (WHO criteria) -no (%)***			
PR^2 ^	16 (24.6%)	6 (100%)	
SD^3^	25 (38.5%)		
PD^4^	24 (36.9%)		
Unmeasurable	16	2	
Unevaluable	1		
***Second-line chemotherapy ***	55 (67.1%)	6 (75.0%)	
***Median follow-up for survivors ***	35.5 months	-	
***Overall survival - mo*. **			
Median	8.2	16	
Interquartile range	(6.8-10.5)	(11.3-26.7)	
***Time to progression - mo*.**			
Median	3.1	8.2	
Interquartile range	(2.5-3.9)	(4.3-21.2)	

^1^Eastern Cooperative Oncology Group, ^2^partial response, ^3^stable disease, ^4^progressive disease

*Relative dose intensity

*Mean of relative dose intensities of cisplatin and fluorouracil. Dose intensity is defined as the amount of drug administered per unit of time, expressed as milligrams per square meter per week. Relative dose intensity is defined as the actual dose intensity relative to the planned dose intensity of each drug.

**Figure 1:**
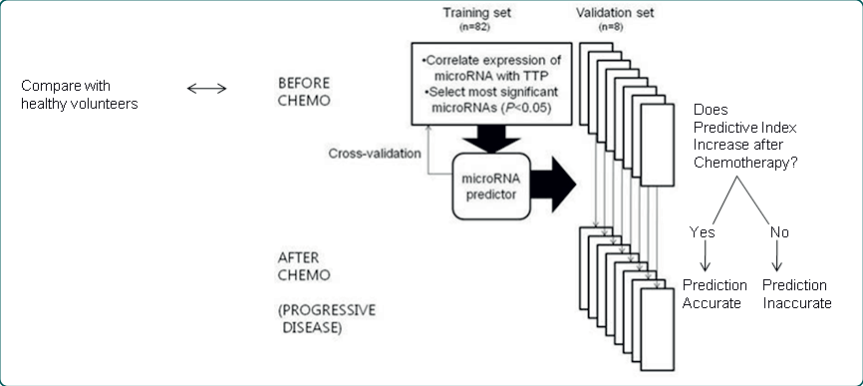
**Study scheme to identify and test miRNAs predictive of resistance to CF**.

We first compared miRNA profiles from the 90 pretreatment samples obtained from gastric cancer patients with the miRNA expression data from 34 normal gastric mucosal biopsy samples obtained from healthy volunteers (Figure 1). To estimate the predictive power of cancer-specific miRNA profiles, class prediction analyses were also performed by randomly dividing the whole sample into two (training and test) subsets at 1-to-1 ratio. Randomization was performed using nQuery Advisor software (version 7.0, Statistical Solutions, Saugus, MA). Then class label of each sample in the test set was predicted for each of 100 random training to test partitions according to compound covariate predictor (CCP), diagonal linear discriminant analysis (LDA), 1- and 3-nearest neighbors (NN), nearest centroid (NC), and support vector machine (SVM). At a feature selection *P*<0.05, the median prediction accuracy in test sets was >90% in all classifiers (91.9%, 90.3%, 90.3%, 93.5%, 93.5%, and 91.9%, for CCP, LDA, 1-NN, 3-NN, NC, and SVM, , respectively), in 100 random training-to-test partitions.

**Figure 2:**
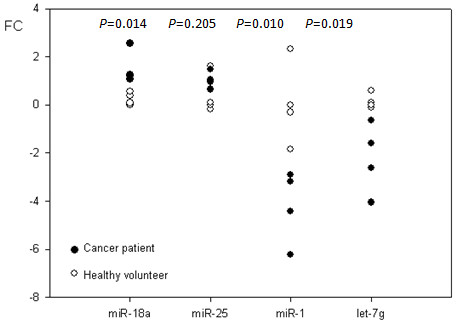
**Validation of miR expression by quantitative real-time reverse transcription polymerase chain reaction (Q-RT-PCR)**. Q-RT-PCR analyses of miR-18a, miR-25, miR-1, and let-7g in 4 normal (*shown in white*) and 4 cancer samples (*shown in black*), confirming over-expression of miR-18a and miR-25 and under-expression of miR-1 and let-7g as observed in the microarray data of the cancer samples. The Student t-test *P *value between 4 normal and 4 cancer samples is shown for each miRNA. Fold change (FC) of -1 indicates a 50% decrease in RNU6-normalized expression of a given miRNA.

Table 2 lists miRNAs that are differentially expressed between the 90 gastric cancer tumors and the 34 normal samples at a feature selection of *P*<0.005. Many miRNAs that are overexpressed in the gastric cancer group belong to the miR-17-92 and 106b-25 clusters, as previously reported [6,7]. Quantitative real-time reverse transcription polymerase chain reaction (Q-RT-PCR) analyses confirmed the differential expression of some of these miRNAs in gastric cancer samples (Figure 2). Although miR-25 was significantly up-regulated by array analysis in the tumors, this did not reach statistical significance by Q-PCR, perhaps due to the limited number of samples that were available for assay. Although a previous study reported that miR-486 is downregulated in gastric cancer, we found expression of miR-486 to be elevated in our set of gastric cancer patients both by microarray and Q-PCR (Additional file 1: Figure S1).

**Table 2:** miRNAs differentially expressed in gastric cancer and normal stomach epithelium.

Overexpressed in gastric cancer	p	FDR	Ratio
hsa-miR-25	< 1e-07	< 1e-07	1.64
hsa-miR-106b	< 1e-07	< 1e-07	1.85
hsa-miR-93	< 1e-07	< 1e-07	1.49
hsa-miR-503	< 1e-07	< 1e-07	2.17
hsa-miR-18a	< 1e-07	< 1e-07	2.27
hsa-miR-224	1.00E-07	2.59E-06	3.85
hsa-miR-451	1.00E-07	2.59E-06	3.23
hsa-miR-18b	2.00E-07	4.60E-06	2.17
hsa-miR-17-5p	2.00E-06	3.60E-05	1.61
hsa-miR-486-5p	3.00E-06	5.18E-05	2.22
hsa-miR-144	9.60E-06	0.000159	5.56
hsa-miR-552	1.03E-05	0.000164	2.38
hsa-miR-425-5p	1.32E-05	0.000195	1.35
hsa-miR-92	1.88E-05	0.000268	1.39
hsa-miR-106a	2.61E-05	0.000347	1.52
hsa-miR-223	2.68E-05	0.000347	2.13
hsa-miR-205	2.98E-05	0.000363	4.76
hsa-miR-196b	4.42E-05	0.000508	1.67
hsa-miR-19a	0.0001836	0.00181	1.69
hsa-miR-191	0.0003112	0.0028	1.27
hsa-let-7i	0.0004468	0.00385	1.20
hsa-miR-185	0.0004764	0.00394	1.32
hsa-miR-769-5p	0.0006683	0.00532	1.37
hsa-miR-196a	0.0008274	0.00646	1.45
hsa-miR-301	0.0009715	0.00745	1.82
hsa-miR-21	0.0012598	0.00948	1.49
hsa-miR-130b	0.0015411	0.0112	1.30
hsa-miR-19b	0.0015959	0.0114	1.39
hsa-miR-424	0.0019249	0.0135	1.52
hsa-miR-484	0.0020451	0.0139	1.33
hsa-miR-767-5p	0.0048511	0.03	1.64
hsa-miR-183	0.0050428	0.0305	1.52
hsa-miR-210	0.0053848	0.0318	1.35
hsa-miR-302c*	0.006328	0.0364	1.41
hsa-miR-520g	0.0070896	0.0402	2.13
hsa-miR-324-5p	0.0095742	0.0497	1.23
hsa-miR-103	0.0095861	0.0497	1.16
hsa-miR-376b	0.0096083	0.0497	1.85
hsa-miR-151	0.0100422	0.0513	1.20
hsa-miR-596	0.011231	0.0556	1.61
hsa-miR-545	0.011422	0.0556	1.69
hsa-miR-221	0.0129139	0.0608	1.27
hsa-miR-20a	0.0133176	0.0619	1.35
hsa-miR-181b	0.0148487	0.0655	1.28
hsa-miR-181d	0.0154891	0.0668	1.16
hsa-miR-623	0.0189476	0.0809	1.43
hsa-miR-519d	0.0220958	0.0915	1.59
hsa-miR-563	0.0229302	0.094	1.37
hsa-miR-505	0.0241657	0.097	1.25
hsa-miR-107	0.0242694	0.097	1.11
hsa-miR-320	0.0282982	0.111	1.20
hsa-miR-96	0.0285699	0.111	1.39
hsa-miR-339	0.0312524	0.12	1.32
hsa-miR-181a	0.0318141	0.121	1.20
hsa-miR-345	0.0322275	0.121	1.19
hsa-miR-20b	0.0325811	0.122	1.28
hsa-miR-33b	0.0339343	0.125	1.64
hsa-miR-135b	0.0352682	0.129	1.59
hsa-miR-431	0.0374687	0.134	1.41
hsa-miR-193a	0.0377098	0.134	1.35
hsa-miR-550	0.0380645	0.134	1.30
hsa-miR-565	0.0446875	0.15	1.20
			
**Underexpressed in gastric cancer**	**p**	**FDR**	**Ratio**
hsa-miR-146a	< 1e-07	< 1e-07	0.39
hsa-miR-133a	< 1e-07	< 1e-07	0.34
hsa-miR-625	< 1e-07	< 1e-07	0.56
hsa-miR-375	< 1e-07	< 1e-07	0.27
hsa-miR-133b	< 1e-07	< 1e-07	0.32
hsa-miR-195	< 1e-07	< 1e-07	0.47
hsa-miR-148a	< 1e-07	< 1e-07	0.47
hsa-miR-1	< 1e-07	< 1e-07	0.27
hsa-miR-26a	< 1e-07	< 1e-07	0.67
hsa-miR-204	2.00E-07	4.60E-06	0.26
hsa-let-7c	7.00E-07	1.53E-05	0.74
hsa-let-7a	9.00E-07	1.86E-05	0.72
hsa-let-7g	1.10E-06	2.17E-05	0.71
hsa-miR-497	1.70E-06	3.20E-05	0.56
hsa-miR-26b	1.28E-05	0.000195	0.58
hsa-miR-145	2.04E-05	0.000282	0.65
hsa-miR-34a	2.89E-05	0.000363	0.75
hsa-miR-143	4.28E-05	0.000506	0.63
hsa-miR-650	9.15E-05	0.00101	0.57
hsa-miR-150	9.25E-05	0.00101	0.49
hsa-miR-768-5p	0.0001037	0.0011	0.65
hsa-let-7d	0.0001302	0.00132	0.76
hsa-miR-203	0.0001311	0.00132	0.52
hsa-miR-29c	0.0002112	0.00203	0.52
hsa-let-7f	0.0002446	0.0023	0.69
hsa-miR-30d	0.0002592	0.00238	0.78
hsa-miR-642	0.0004345	0.00383	0.62
hsa-miR-30c	0.0004556	0.00385	0.75
hsa-miR-155	0.0004998	0.00406	0.66
hsa-miR-34b	0.0013651	0.0101	0.64
hsa-miR-551b	0.0019808	0.0137	0.53
hsa-miR-28	0.0027537	0.0184	0.85
hsa-let-7e	0.0034793	0.0227	0.84
hsa-let-7b	0.0035019	0.0227	0.85
hsa-miR-212	0.0039061	0.0249	0.76
hsa-miR-564	0.0047906	0.03	0.72
hsa-miR-770-5p	0.0050814	0.0305	0.71
hsa-miR-30b	0.0060842	0.0355	0.76
hsa-miR-30a-5p	0.0077597	0.0434	0.80
hsa-miR-199b	0.0083572	0.0461	0.67
hsa-miR-125a	0.0085563	0.0466	0.77
hsa-miR-621	0.0093423	0.0497	0.69
hsa-miR-31	0.0106862	0.054	0.66
hsa-miR-365	0.0113404	0.0556	0.78
hsa-miR-381	0.0123061	0.0592	0.70
hsa-miR-626	0.0128738	0.0608	0.78
hsa-miR-127	0.0138033	0.0635	0.69
hsa-miR-660	0.0142991	0.0651	0.75
hsa-miR-342	0.0146193	0.0655	0.75
hsa-miR-146b	0.0148729	0.0655	0.77
hsa-miR-361	0.0152056	0.0663	0.86
hsa-miR-489	0.0191692	0.081	0.71
hsa-miR-29a	0.0204334	0.0854	0.79
hsa-miR-95	0.0243644	0.097	0.54
hsa-miR-567	0.0265025	0.104	0.54
hsa-miR-152	0.0376121	0.134	0.78
hsa-miR-429	0.0378151	0.134	0.65
hsa-miR-200b	0.0396617	0.138	0.75
hsa-miR-504	0.0412648	0.142	0.63
hsa-miR-668	0.041717	0.143	0.77
hsa-miR-186	0.0437991	0.149	0.83
hsa-miR-135a	0.0468793	0.157	0.58
hsa-miR-485-5p	0.047683	0.158	0.82

### Identification of a miRNA signature for resistance to CF therapy

Time to progression (TTP), not radiographic response, was used as the clinical indicator for chemotherapy response, primarily because we wished to include patients who did not have quantifiable disease using standard imaging modalities. To define a from 82 samples as the training set to develop a predictor (Figure 1). These 82 pretreatment samples were collected from patients who did not undergo second biopsies. Fifty-eight miRNAs were significantly correlated with the TTP of these 82 patients (feature selection *P *value<0.05) (Table 3). The overexpression of 30 miRNAs was associated with delayed TTP whereas the overexpression of 28 miRNAs was associated with a more rapid TTP. Six *miRNAs that were associated with chemoresistance, including miR-518f*, miR-520a, miR-520d*, miR-519e*, miR-363*, and miR-517*, whereas no *miRNAs were associated with chemosensitivity.

**Table 3:** miRNAs whose expression is associated with chemosensitivity or chemoresistance.

miRNAa whose expression is associated with chemoresistance	p	FDR	Hazard Ratio
hsa-miR-526a	0.0000	0.0103	1.482
hsa-miR-122a	0.0002	0.0379	1.545
hsa-miR-518f*	0.0004	0.0537	1.298
hsa-miR-591	0.0007	0.0598	1.492
hsa-miR-524-3p	0.0010	0.0682	1.268
hsa-miR-320	0.0013	0.0701	1.865
hsa-miR-520a*	0.0014	0.0701	1.252
hsa-miR-183	0.0031	0.119	1.39
hsa-miR-516-5p	0.0034	0.119	1.306
hsa-miR-629	0.0036	0.119	1.42
hsa-miR-595	0.0043	0.119	1.858
hsa-miR-640	0.0054	0.132	1.3
hsa-miR-520d*	0.0063	0.143	1.326
hsa-miR-519e*	0.0091	0.164	1.24
hsa-miR-363*	0.0096	0.166	1.407
hsa-miR-513	0.0137	0.193	1.347
hsa-miR-328	0.0163	0.211	1.736
hsa-miR-519a	0.0170	0.211	1.118
hsa-miR-185	0.0189	0.217	1.697
hsa-miR-658	0.0200	0.223	1.532
hsa-miR-517*	0.0218	0.226	1.305
hsa-miR-515-5p	0.0349	0.301	1.145
hsa-miR-519c-5p	0.0368	0.304	1.157
hsa-miR-661	0.0392	0.315	1.437
hsa-miR-182	0.0416	0.315	1.408
hsa-miR-206	0.0417	0.315	1.606
hsa-miR-193b	0.0419	0.315	1.433
hsa-miR-601	0.0436	0.317	1.599
			
**miRNAS whose expression is associated with chemosensitivity**	**p**	**FDR**	**Hazard Ratio**
hsa-miR-195	0.0007	0.0598	0.593
hsa-miR-146b	0.0016	0.0733	0.565
hsa-miR-26b	0.0037	0.119	0.686
hsa-miR-374	0.0042	0.119	0.84
hsa-miR-199b	0.0051	0.132	0.729
hsa-miR-132	0.0068	0.143	0.62
hsa-miR-140	0.0069	0.143	0.759
hsa-miR-487b	0.0088	0.164	0.679
hsa-let-7g	0.0091	0.164	0.539
hsa-miR-340	0.0103	0.171	0.82
hsa-miR-155	0.0109	0.174	0.704
hsa-miR-95	0.0115	0.176	0.856
hsa-miR-186	0.0137	0.193	0.662
hsa-miR-130a	0.0140	0.193	0.72
hsa-miR-342	0.0151	0.202	0.685
hsa-miR-577	0.0173	0.211	0.804
hsa-miR-128b	0.0184	0.217	0.701
hsa-miR-146a	0.0209	0.226	0.776
hsa-miR-16	0.0214	0.226	0.698
hsa-miR-503	0.0241	0.243	0.721
hsa-miR-224	0.0259	0.25	0.853
hsa-miR-223	0.0259	0.25	0.794
hsa-miR-128a	0.0294	0.276	0.704
hsa-miR-181b	0.0300	0.276	0.668
hsa-let-7f	0.0312	0.281	0.725
hsa-miR-1	0.0339	0.298	0.839
hsa-miR-421	0.0367	0.304	0.738
hsa-miR-127	0.0404	0.315	0.783
hsa-miR-34c	0.0435	0.317	0.74
hsa-miR-497	0.0493	0.351	0.769

**Figure 3:**
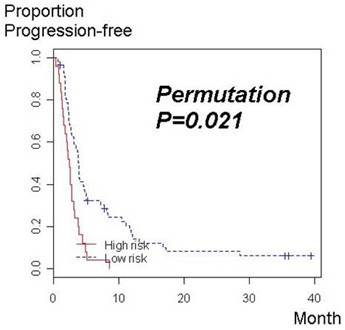
**Kaplan-Meier curves for the time to progression (TTP) of 2 risk groups stratified according the expression of 58 miRNAs correlated with TTP at a feature selection P < 0.05**. The association of miRNA expression data to TTP was statistically significant (permutation *P *value for log-rank statistics of cross-validated Kaplan-Meier curves = 0.021).

Figure 3 depicts a Kaplan-Meier curve for risk groups stratified by these 58 miRNAs. A permutation significance level for the log-rank statistic of leave-one-out cross-validated Kaplan-Meier curves was 0.021, suggesting that the association between miRNA expression data to TTP is statistically significant.

### Expression of the chemoresistance signature correlates with the evolution of chemoresistance in tumors that were previously chemosensitive

The 58 miRNA signature identified above is predictive for identifying patients who are or are not likely to respond favourably to CF therapy. We postulated that patients who initially demonstrated a favourable 58 miRNA expression signature would switch to an unfavourable expression signature at the time they developed resistance to CF therapy. In order to test this possibility as a proof-of-principle, 8 pairs of test set samples (endoscopically obtained pre- and post-treatment) were collected from 8 patients who initially demonstrated a clinical response to CF treatment but who eventually showed progressive disease at which time a second endoscopic biopsy was taken. As shown in Table 4 all of the 8 pretreatment samples from the clinical responders were correctly predicted by the 58-miRNA predictor to be in the low-risk group (accuracy, 100%). Notably, 6 out of 8 pairs were correctly identified for chemosensitivity (*i.e*., posttreatment samples were assigned a higher predictive index for chemotherapy response than pretreatment samples, and therefore, predicted to be more resistant to therapy) (accuracy, 75%). When the same prediction was performed using the feature selection *P *value of 0.01, the prediction result remained the same (Table 4).

**Table 4:** Prediction for chemoresistance in the proof-of-principle test set

*Sample ID*	*Feature selection P < 0.05*				*Feature selection P < 0.01*		
	Predictive Index Percentile^1^	Prediction For Pretreatment Sample^2^	Overall Prediction^3^		Predictive Index Percentile	Prediction for Pretreatment Sample	Overall Prediction
1 pre	39%	low	incorrect		35%	low	incorrect
1 post	22%				24%		
							
2 pre	48%	low	correct		49%	low	correct
2 post	79%				77%		
							
3 pre	55%	low	correct		46%	low	correct
3 post	73%				77%		
							
4 pre	13%	low	correct		24%	low	correct
4 post	72%				73%		
							
5 pre	31%	low	correct		33%	low	correct
5 post	48%				46%		
							
6 pre	13%	low	correct		27%	low	correct
6 post	16%				66%		
							
7 pre	7%	low	correct		12%	low	correct
7 post	66%				57%		
							
8 pre	52%	low	incorrect		50%	low	incorrect
8 post	17%				24%		

^1^The predictive index was computed for each sample by this supervised principal component method, where a high value of the predictive index corresponds to a rapid progression after chemotherapy (*i.e*., short TTP). If the predictive index of a sample in the test set corresponded to the median predictive index of the training set, the sample was assigned a 50% predictive index.

^2^The risk was predicted low, if predictive index percentile of the pretreatment sample was less than 67%

^3^The prediction was considered correct if post-treatment samples were assigned a higher predictive index than pre-treatment samples.

## Discussion

This study has utilized a prospective approach to identify a miRNA signature for gastric cancer vs. normal stomach epithelium and a miRNA signature that predicts response to standard CF therapy.

Since routine histopathology techniques sometimes do not lead to a definitive diagnosis of gastric cancer, the addition of a miRNA signature from such patient samples may improve the accuracy of a diagnosis of gastric cancer. In previous miRNA microarray studies of gastric cancer, control tissues were obtained from regions of the stomach of gastric cancers patients that were determined to be histologically normal and not from stomach tissue of healthy normal volunteers [6,7]. Since molecular abnormalities are often found in histologically normal-appearing tissue adjacent to tumor tissue, we chose to obtain control tissues from endoscopic biopsy samples from normal, cancer-free volunteers. Most of the differentially expressed miRNAs reported to be characteristic of gastric cancer in previous microarray studies [6,7] were also identified within the gastric cancer signature in our current analyses. However, in addition to these previously reported miRNAs in gastric cancer, we additionally identified potential tumor suppressor miRNAs (at *P*<0.05 [6]and *P*<0.01[7], including miR-1[8,9] and let-7 [10] that we found to be underexpressed in gastric cancer (at *P*<0.001) (Table 2). Interestingly, Oh et al found expression of miR-486 to be reduced in many gastric cancers, in some cases, associated with a genomic loss of that region [11]. We found miR-486 to be overexpressed in our gastric cancer cohort by both microarray and Taqman PCR analysis **(**Additional file 1: Figure S1). It is possible that this difference in results is due to very different patient populations studied.

In this study, we report for the first time to our knowledge, a miRNA predictor for response to CF therapy. The 58 miRNA signature that provides an index for assessing potential response to CF therapy may be useful in stratifying patients into a group that should receive standard therapy and a group that will likely not benefit from such therapy and should be placed on a different therapeutic trial. Several of the 58 miRNAs we identified in Table 2 that are associated with TTP are consistent with published reports relating their expression with chemoresistance and tumor biology. Prominent among the upregulated miRNAs associated with a prolonged TTP (defined by a hazards ratio <1) were miRNAs that have been shown to induce apoptosis in gastric and other cancer cells, such as miR-16, let-7g, miR-181, miR-342, miR-1, and miR-34 [8,12-18]. miR-16 augments apoptosis induction by nutlin and genistein[12], and modulates multidrug resistance of human gastric cancer cells[13].

Overexpression of let-7c or let-7g has been shown to decrease expression of Bcl-xL in Huh7 and HepG2 cell lines [14]. Let-7g and miR-181b are positively correlated with clinical responsiveness of colon cancer to S-1, an oral fluorouracil [4]. miR-181a and miR-181b have been shown to function as tumor suppressors which trigger growth inhibition, induce apoptosis and inhibit invasion in glioma cells[15]. Reconstitution of hsa-miR-342 in the colorectal cancer cell line HT-29 induces apoptosis [16]. miR-1 sensitizes lung cancer cells to doxorubicin-induced apoptosis [8]. Ectopic miR-34 expression induces apoptosis, cell-cycle arrest or senescence in normal and tumor cells [17]. Thus, overexpression of these pro-apoptotic miRNAs in primary tumors appears to be a highly consistent feature of patients who benefits from CF.

Interestingly, we identified six *miRNAs that were associated with chemoresistance, including miR-518f*, miR-520a, miR-520d*, miR-519e*, miR-363*, and miR-517*, whereas no miRNAs were associated with chemosensitivity. Only one miR, miR-302*, was identified in the gastric cancer miR signature. miR*s are considered to be passenger strands that are thought to normally be degraded from the pre-miR which results in the mature 22 nt strand that enters the RISC complex. The functions of *miRNAs remain unclear, although it is possible that they result from impaired processing of pre-miRNAs (Tchernitsa et al J of Pathology, 2010) or may play a role in targeting mRNA translation (Gu and Lu, Plos One, 2010).

We also observed that while 21 miRNAs were found in common between the GC and chemoresistance miRNA signatures, 37 miRNAs were unique to the chemosensitivity signature.

Analysis of the sample pairs pre- and post-treatment from 8 patients who initially responded to CF therapy but later became resistant to therapy served as a proof-of-principle for demonstrating that the predictive index of the 58 miRNA signature would switch from a favourable index (at the pre-treatment stage) to an unfavourable index (post-treatment when resistance developed). Unfortunately, it was not possible to obtain additional matched pairs of samples from similar patients to provide a more robust statistical analysis. Nevertheless, the results are consistent with a model of clonal selection of pre-existing resistant tumors cells residing within the primary tumor.

According to the conventional clonal selection model for the development of acquired resistance to chemotherapy resistance, resistance of initially responsive tumors develops due to the selective outgrowth of chemoresistant clones that already exist within the tumor [18]. Given that a rapid TTP specifically indicates an intrinsic resistance to chemotherapy [19], the 58 miRNAs whose expression levels are correlated with a short TTP may represent chemoresistance-related miRNAs already present in the majority of the tumor cells in the primary tumor. However, primary tumors that appear not to express this miRNA signature of resistance, initially respond to therapy until preexisting, resistant cells selectively grow despite CF therapy. At the time a sample is obtained when resistance is observed, the bulk of the tumor expresses the unfavourable, chemoresistant miRNA signature. Given that resistance in most of these patients develops over a relatively short period of time (months, not years), it seems unlikely that resistance results from the accumulation of multiple individual genetic changes.

The results of this study provide important new data and miRNA signatures, especially predicting response to CF therapy and regarding the emergence of tumor resistance. However, larger studies need to be conducted in the future to further validate these findings and determine whether they can be applied in a clinical setting.

## Conclusions

Although limited by the small sample size of the validation set, this study identifies miRNAs that may comprise a clinically relevant signature for intrinsic resistance of gastric cancer to CF and suggests that these miRNAs were selected for during the development of acquired chemoresistance. Since this miRNA predictor may possibly provide a useful guide to personalized chemotherapy in the future, it warrants further investigation and validation in large prospective studies.

## Methods

### Patient enrolment and treatment

Tissue samples were collected at the hospital of Korean National Cancer Center by endoscopy from 2001 to 2006 under a protocol approved by the Institutional Review Board (IRB) of the National Cancer Center Hospital in Goyang, Korea. All patients and volunteers signed IRB-approved informed consent forms. Eligibility for enrolment into the study included the following parameters: 1) age ≥ 18 years; 2) histologically confirmed gastric adenocarcinoma; 3) documented distant metastasis; 4) no previous or concomitant malignancies other than gastric cancer; 5) no prior chemotherapy, either adjuvant or palliative; and 6) adequate function of all major organs. 34 healthy volunteers underwent gastroscopy for routine screening for gastric cancer and had normal gastric mucosa by histology. There was no gastritis among the 34 healthy volunteers.

This miRNA study has been performed as a parallel study to a study of mRNA expression analysis[20] designed to identify mRNA predictors of chemoresistance. Ninety pre-treatment biopsy samples collected from 2001 to 2006 were analyzed in this miRNA study. After an initial endoscopic biopsy, all of the 90 patients were treated with cisplatin (60 mg/m^2^, D1) in combination with either fluorouracil (1 g/m^2 ^for 5 days; n = 88) or capecitabine (Xeloda; Roche; 1,250 mg/m^2 ^BID for 2 weeks; n = 2) every 3 weeks. Clinical responders were asked to undergo the second endoscopy at the time progressive disease (PD) was observed according to World Health Organization (WHO) criteria. The following two criteria were used to define clinical responders: 1) patients whose tumors demonstrated more than a 50% decrease in the sum of the products of the two largest perpendicular diameters of measurable lesions for at least 4 weeks; or 2) patients who did not have measurable disease at presentation and had a dramatic decrease in pleural effusion/ascites for at least 4 weeks [21]. Post-treatment miRNA microarray data could be obtained from samples collected when chemoresistance developed (PD) in 8 clinical responders. Post-treatment samples were collected at least 2 weeks after the last dose of the fluorouracil, and before second-line chemotherapy was started, in order to avoid any acute drug effects on influencing the expression profile. For these 8 clinical responders, pre- and post-treatment samples (which were collected at the time of progressive disease) represent chemosensitive and chemoresistant tumors, respectively. Pretreatment samples from the remaining 82 patients were used to identify a miRNA predictor for chemotherapy response. This predictor was applied to 8 sample pairs collected from the same patients pre- and post-treatment. The prediction was considered correct if post-treatment samples were assigned a higher predictive index for chemoresistance than the pre-treatment samples. Biopsy samples were similarly collected from 34 healthy volunteers.

Tissue samples containing at least 50% tumor cells were processed for RNA as previously described [22]. The extracted RNAs were assayed using the Agilent Bioanalyzer 6000 Total RNA assay and the Nanodrop spectrophotometer following manufacturer's protocols. 500 ng of total RNA was subjected to a custom miRNA microarray. A mixture of total RNA isolated from three gastric cancer cell lines (SNU-601, SNU-638, and AGS) was used as the reference RNA for competitive hybridization.

### Microarray experiment

#### miRNA microarray design

The Laboratory of Molecular Technology (LMT)_miRNA_v2 microarray was designed using the Sanger miR9.0 database (http://microrna.sanger.ac.uk) and manufactured as a custom-synthesized 8 × 15 K microarrays (Agilent Technologies, San Jose, CA). There are a total of 4,361 miRNA entries in the miR9.0 database. Some of the miRNAs have exact sequences from different species. We collapsed the database to 1,667 unique mature miRNA sequences across all species, including human, mouse, rat, etc. The mature miRNA sequences were incorporated into 60-mer long oligonucleotide probes with a linker sequence on the 3' end to separate the miRNA sequences away from the glass slide surface. The linker sequence was a proprietary sequence from Agilent that has minimal homology to any sequence in the GenBank. Each mature miRNA is represented by + and - (reverse complement) strand sequences. This enables the microarray to be used with different labeling protocols. Depending on the protocol, one of the probes can also serve as a negative control. Each probe has 4 replicates within each microarray, providing technical replicates for measuring consistency and performance of the microarray. In summary, each unique mature miRNA is represented by 8 probes (4 + strand and 4 - strand). A total of 3,556 unique LMT seq IDs (miRNA, positive and negative controls, +/- strand) were on the microarray, each with 4 replicates. Advantages of the microarray include high sensitivity (requiring < 1 microgram of total RNA) and high reproducibility (CV = 1%).

#### Validation of the LMT miRNA platform

Only 1 microgram of total RNA containing miRNAs was required for the microarray. The sensitivity of the LMT miRNA microarray platform was compared with other miRNA arrays (Additional file 2: Table S1). In an experiment comparing two reference RNAs - Ambion brain and liver RNAs containing miRNAs - the LMT microarray detected similar percentages of miRNAs as compared to other commercial miRNA microarray platforms. It was more sensitive than the Agilent miRNA microarray but less sensitive than the Affymetrix FT-HSR miRNA microarray and the Taqman miRNA card.

To test the global specificity of the microarray, we compared the results between platforms of miRNA expression measured using two commercial reference RNAs containing microRNAs for brain and liver (Ambion). The fold-changes observed between these two samples across the different microRNAs microarray platforms were determined. A high degree of concordance was observed between the microRNAs identified by the LMT miRNA microarray vs the Agilent miRNA microarray and the Affymetrix FT-HSR miRNA microarray (Additional file 3: Figure S2). In addition, a high degree of correlation was observed between the LMT miRNA microarray and the Affymetrix FT-HSR microRNA array (0.707) (as well as the Agilent miRNAmicroarray (0.882) (Additional file 4: Figure S3).

#### miRNA determinations using the LMT miRNA microarray

The total RNAs containing the microRNAs were labeled using the miRCURY™ LNA microRNA Array Power Labeling kit (Exiqon Inc, Woburn, MA). The 3'-end of the total RNA was enzymatically labeled with the Hy3 and/or Hy5 fluorescent dye (Exiqon), and the labeled RNA was hybridized onto the microarrays without the need for column purification. The washed and dried slides were scanned using the Agilent scanner. The Feature Extraction program was used to extract the spot intensities. All microarray data has been deposited at GEO and is available upon publication. Reviewer access: http://www.ncbi.nlm.nih.gov/geo/query/acc.cgi?token=ftixhsoiemwgyfi&acc=GSE30070

### miRNA quantitative RT-PCR (Q-RT-PCR)

Q-RT-PCR reaction was performed for miR-18a, miR-25, miR-1, and let-7g, and miR-486, using the miScript PCR system (QIAGEN, Valencia, CA) in duplicate reactions in a 96-well plate. Cycle threshold (C_t_) values of miRNA expression were normalized to RNU6 by subtraction. C_T _values were determined, where C_T _represents the threshold cycle or the PCR cycle number at which an increase in reporter fluorescence crosses a threshold significantly above the baseline signal. For data normalization, RNU6 was selected as the reference endogenous control miRNA. Relative quantification of each mRNA was achieved by first normalizing the specific mRNA C_T _values to one reference C_T _value, RNU6, then comparing the test samples to control samples. Specifically, the ΔC_T _-Sample value was calculated as ΔC_T _Sample = avg. C_T _Sample - avg. C_T _Reference, then the ΔΔC_T _Test to Control =Δ C_T _Sample Test - ΔC_T _Sample Control. RT-PCR expression level was calculated by raising 2 to the power of - ΔΔC_T _Test to Control, and compared between cancer and normal samples using the Student *t*-test.

### Survival analysis

miRNA data were analyzed using BRB-ArrayTools (version 3.6, National Cancer Institute, http://linus.nci.nih.gov/BRB-ArrayTools.html) [23]. Array data were log-transformed and normalized using Lowess smoother. The survival analysis tool identified genes whose expression is correlated with TTP by fitting a proportional hazards model relating survival to the expression of each miRNA. *P *values are calculated for each gene to test the hypothesis that survival time is independent of the expression level for that gene. Time to progression (TTP) was used as the clinical indicator for chemotherapy response. TTP was calculated from the initiation of chemotherapy to the onset of progressive disease. In patients without any measurable lesions, time to progression was measured to the time when a change in therapy was required because unmeasurable lesions (such as ascites) unequivocally progressed [24]. Prediction of chemotherapy response was performed using the survival risk prediction algorithm of BRB-ArrayTools. The survival risk groups were constructed using a predictive index based upon the supervised principal component method of Bair and Tibshirani [25]. The predictive index was based on the weighted average of the log intensities of the discriminatory miRNAs using a proportional hazards regression on the first two principal components of the log intensities of those miRNAs. The predictive index was computed for each sample by this supervised principal component method, where a high value of the predictive index corresponds to a rapid progression after chemotherapy (*i.e*., short TTP). For instance, if the predictive index of a sample in the test set corresponded to the median predictive index of the training set, the sample was assigned a 50% predictive index. We specified the number of risk groups as 2 (high and low) and the predictive index percentile for delineating the two risk groups as 67%, since our low risk group included 63.1% of patients with a clinical benefit from therapy (partial response and stable disease) and 36.9% of patients in the high risk group with progressive disease in the training set.

The survival risk group prediction algorithm of BRB-ArrayTools also provides an assessment of whether the association of miRNA expression data to survival data is statistically significant. A log-rank statistic was computed for the cross-validated Kaplan-Meier curves. For each random re-shuffling, BRB-ArrayTools repeats the process, creates new cross-validated Kaplan-Meier curves, and computes the log-rank statistic for the random shuffling. This provides a null-distribution of the log-rank statistic created in this way. The tail area of this null distribution beyond the value LR_d _obtained for the real data is the permutation significance level for testing the null hypothesis that there is no relation between the expression data and survival. This permutation significance level was considered significant if it was less than 0.05.

## Competing interests

The authors declare that they have no competing interests.

## Authors' contributions

CHK designed and developed the miRNA microarrays, performed microarray experiments, analyzed the miRNA data analysis and wrote the manuscript. HKK coordinated the collection of all clinical samples, performed the statistical analyses of the miRNA data, developed the gastric cancer miRNA and chemoresistance signatures and contributed to the writing of the manuscript. RLR performed the miRNA microarray experiments in conjunction with CHK. JK and ETL contributed to sample preparation for array analysis. OA performed Q-PCR studies. IJC collected patient samples. DM contributed to the design of the miRNA microarrays and study design. JG contributed to the study design, data analysis and contributed to the writing of the manuscript. All authors read and approved the final manuscript.

## Pre-publication history

The pre-publication history for this paper can be accessed here:

http://www.biomedcentral.com/1755-8794/4/79/prepub

## Supplementary Material

Additional file 1 **Supplemental Figure **1**: Validation of miR expression by quantitative real-time reverse transcription polymerase chain reaction (Q-RT-PCR)**. Q-RT-PCR analyses of miR-486 in 12 normal (*circles*) and 7 cancer samples (*squares*), confirming over-expression of miR-486 as observed in the microarray data of the cancer samples. Click here for file

Additional file 2 **Supplemental Table **1**: Cross platform comparisons of miRNA expression**. Comparing the data for detection of microRNAs in the same two tissue samples (brain and liver), the ABI Taqman Array MicroRNA Card platform and the Affymetrix/FlashTagHSR platforms demonstrated the highest percent present calls and were nearly identical on their respective platforms, followed by our LMT miRNA microarray and the Agilent platform. Click here for file

Additional file 3 **Supplemental Figure **2**: Concordance of matching probes between array platforms**. We compared the LMT, Affymetrix FlashTag HSR and Agilent microRNA microarray platforms to one another looking at matching direction of the fold change (up or down). Of the 3 planar microarray platforms, the Affymetrix FlashTag HSR had the highest number of combined up and down regulated miRNA at 111 followed by Agilent with 101 and LMT with 78. Ninety-one, 91, up and down -regulated miRNAs were shared between Agilent and Affymetrix FlashTag HSR, 74 were shared LMT and Affymetrix FlashTag HSR, and 74 were shared between LMT and Agilent. Click here for file

Additional file 4 **Supplemental Figure **3**: Correlations between miRNA array platforms**. To study the correlation of the absolute fold changes between each microarray platform, the fold change data (n = 140) was Log 2 transformed, plotted and the Pearson correlation, r, calculated between platforms (Figure 3). We first compared all of the platforms to our LMT legacy platform to determine which of the commercial platforms correlates best with our reference platform. The Agilent platform demonstrated the highest correlation to our LMT array data (r = 0.882) based on absolute fold change. Click here for file
