# Supplementary figures and images for: miRNA signature associated with outcome of gastric cancer patients following chemotherapy
Source: BMC Med Genomics. 2011 Nov 23;4:79. doi: 10.1186/1755-8794-4-79 (PMC3287139; doi:10.1186/1755-8794-4-79)

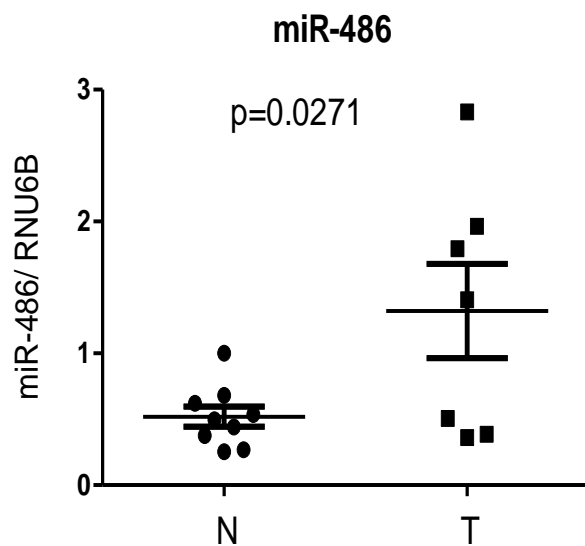

Supplemental Figure 1.

Supplement: Additional file 1 — Supplemental Figure 1: Validation of miR expression by quantitative real-time reverse transcription polymerase chain reaction (Q-RT-PCR). Q-RT-PCR analyses of miR-486 in 12 normal (circles) and 7 cancer samples (squares), confirming over-expression of miR-486 as observed in the microarray data of the cancer samples. [file 1755-8794-4-79-S1.PDF]

Supplementary Figure 2

p<0.05, n=140

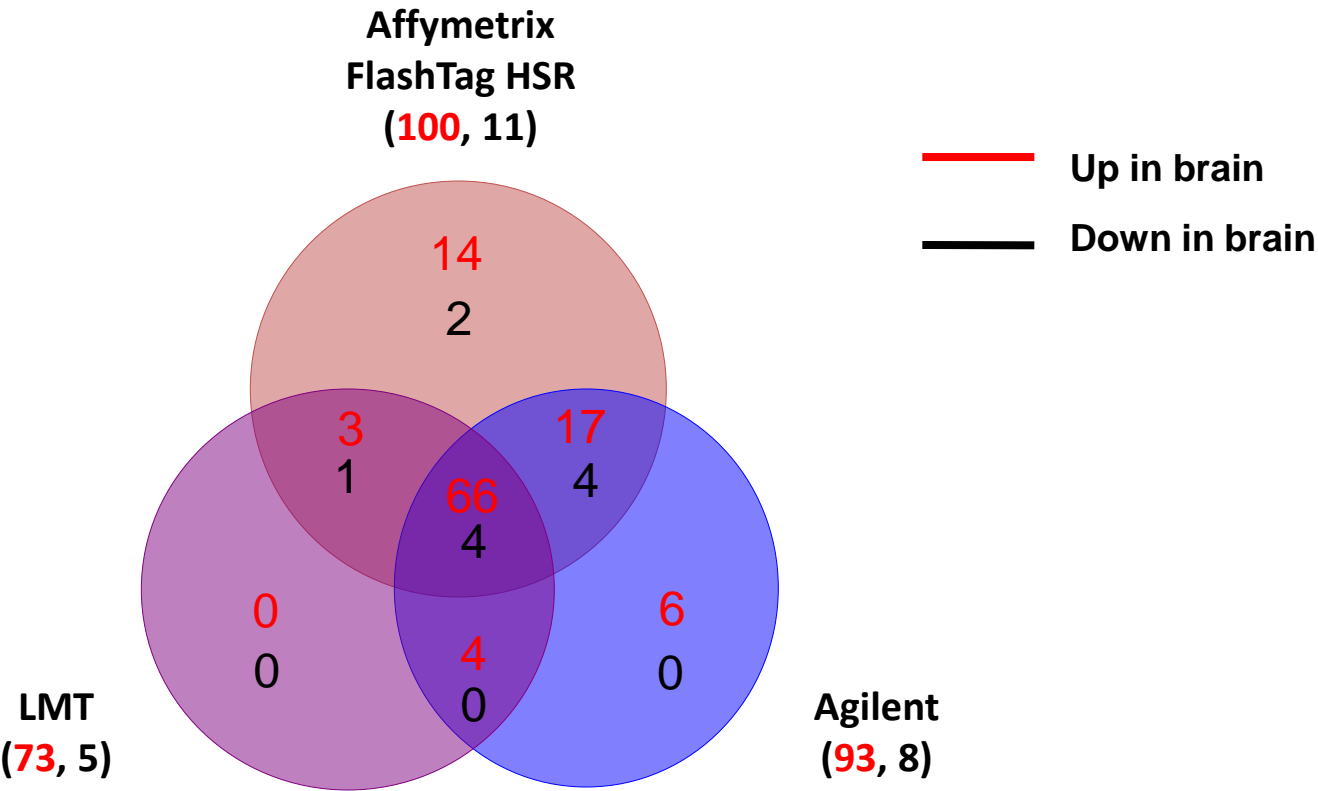

Supplement: Additional file 3 — Supplemental Figure 2: Concordance of matching probes between array platforms. We compared the LMT, Affymetrix FlashTag HSR and Agilent microRNA microarray platforms to one another looking at matching direction of the fold change (up or down). Of the 3 planar microarray platforms, the Affymetrix FlashTag HSR had the highest number of combined up and down regulated miRNA at 111 followed by Agilent with 101 and LMT with 78. Ninety-one, 91, up and down -regulated miRNAs were shared between Agilent and Affymetrix FlashTag HSR, 74 were shared LMT and Affymetrix FlashTag HSR, and 74 were shared between LMT and Agilent. [file 1755-8794-4-79-S3.PDF]

Supplementary  
Figure 3

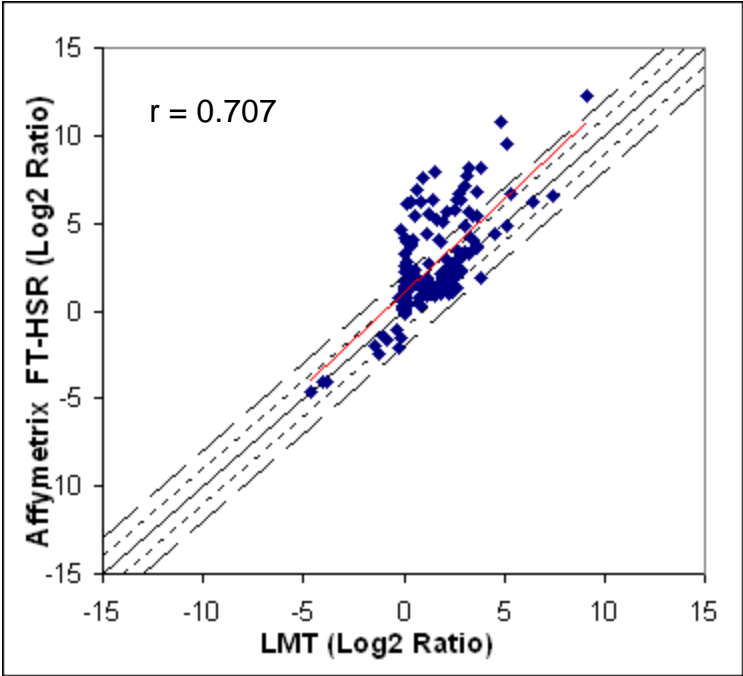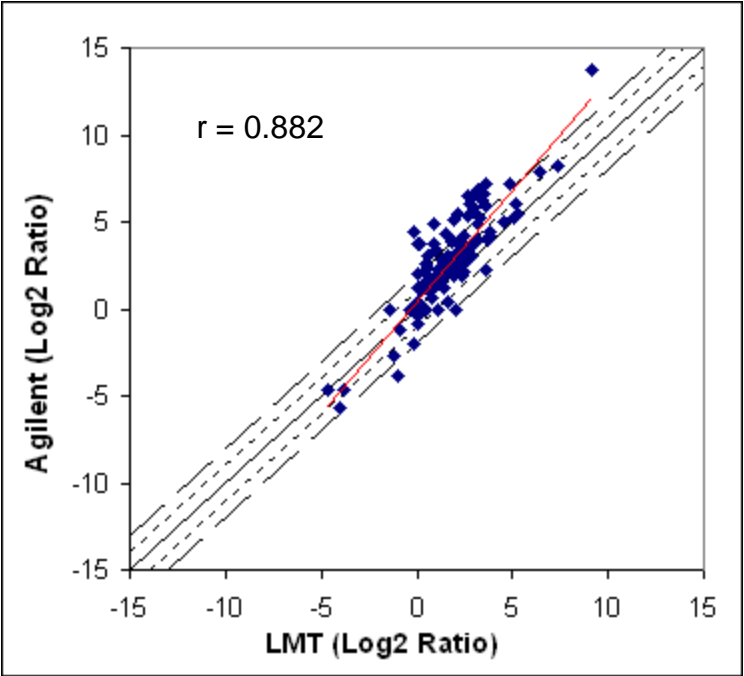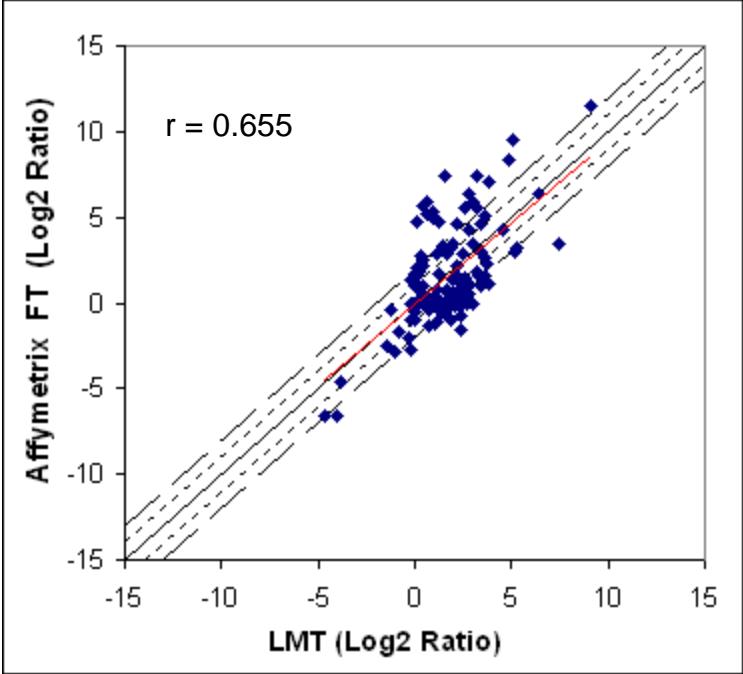

Supplement: Additional file 4 — Supplemental Figure 3: Correlations between miRNA array platforms. To study the correlation of the absolute fold changes between each microarray platform, the fold change data (n = 140) was Log 2 transformed, plotted and the Pearson correlation, r, calculated between platforms (Figure 3). We first compared all of the platforms to our LMT legacy platform to determine which of the commercial platforms correlates best with our reference platform. The Agilent platform demonstrated the highest correlation to our LMT array data (r = 0.882) based on absolute fold change. [file 1755-8794-4-79-S4.PDF]
