# Supplementary material for: miRNA signature associated with outcome of gastric cancer patients following chemotherapy
Source: BMC Med Genomics. 2011 Nov 23;4:79. doi: 10.1186/1755-8794-4-79 (PMC3287139; doi:10.1186/1755-8794-4-79)
Supplement: Additional file 2 — Supplemental Table 1: Cross platform comparisons of miRNA expression. Comparing the data for detection of microRNAs in the same two tissue samples (brain and liver), the ABI Taqman Array MicroRNA Card platform and the Affymetrix/FlashTagHSR platforms demonstrated the highest percent present calls and were nearly identical on their respective platforms, followed by our LMT miRNA microarray and the Agilent platform. [file 1755-8794-4-79-S2.PDF]

Supplementary Table 1

Platform	Content Sanger miRBase	Release Date	# Human miRNAs	Recommended Input RNA (ng)	Tested Input RNA (ng)	Brain Detected miRNAs	Brain % Present miRNAs	Liver Detected miRNAs	Liver % Present miRNAs
LMT	Version 9	January 2007	372	1000	1000	156	42%	105	28%
Agilent	Version 10	January 2009	723	≥100	100	213	29%	122	17%
Affymetrix - Former FlashTag	Version 11	March 2009	847	500-3000	1000	312	37%	252	30%
Affymetrix - FlashTag HSR	Version 11	February 2010	847	≥100	300	411	49%	319	38%
ABI Taqman miRNA Array	Version 10	January 2009	644	≥500	500	319	50%	236	37%

Method for determine present call:

LMT = 2x over background

Agilent = Agilent Boolean

Affymetrix = Affymetrix miRNA QC software

ABI Taqman = Ct < 32 (ABI)
